# A generalized motion compensated compressed sensing scheme for highly accelerated myocardial perfusion MRI

**DOI:** 10.1186/1532-429X-16-S1-W23

**Published:** 2014-01-16

**Authors:** Sajan Goud Lingala, Edward V DiBella, Mathews Jacob

**Affiliations:** 1 The University of Iowa, Iowa City, Iowa, USA; 2 University of Utah, Salt lake City, Utah, USA

## Background

Compressed sensing (CS) based myocardial perfusion MRI methods that promote sparsity in temporal transform domains such as temporal Fourier (x-f), temporal PCA (x-PCA), temporal total variation (x-TV) have shown promise to accelerate breath held scans [Otazo et al,10, Pedersen et al,09, Adluru et al,07]. However the performances of these schemes can degrade in the presence of motion if the sparse representations in these transforms are significantly disturbed. In this work, we propose to address this challenge by jointly estimating and compensating for the motion during the CS reconstruction (MC-CS). The proposed scheme employs a variable splitting based optimization strategy [Lingala et al 2011] to enable joint motion estimation along with reconstruction. Unlike existing MC-CS methods, the novelties enabled by this optimization are a generalized formulation capable of handling any temporal sparsifying transform, no requirement of fully sampled prescans or navigators for motion estimation. We compare the performance of the MC-CS method with three different sparsifying transforms on free breathing myocardial perfusion data.

## Methods

**Figure 1:**
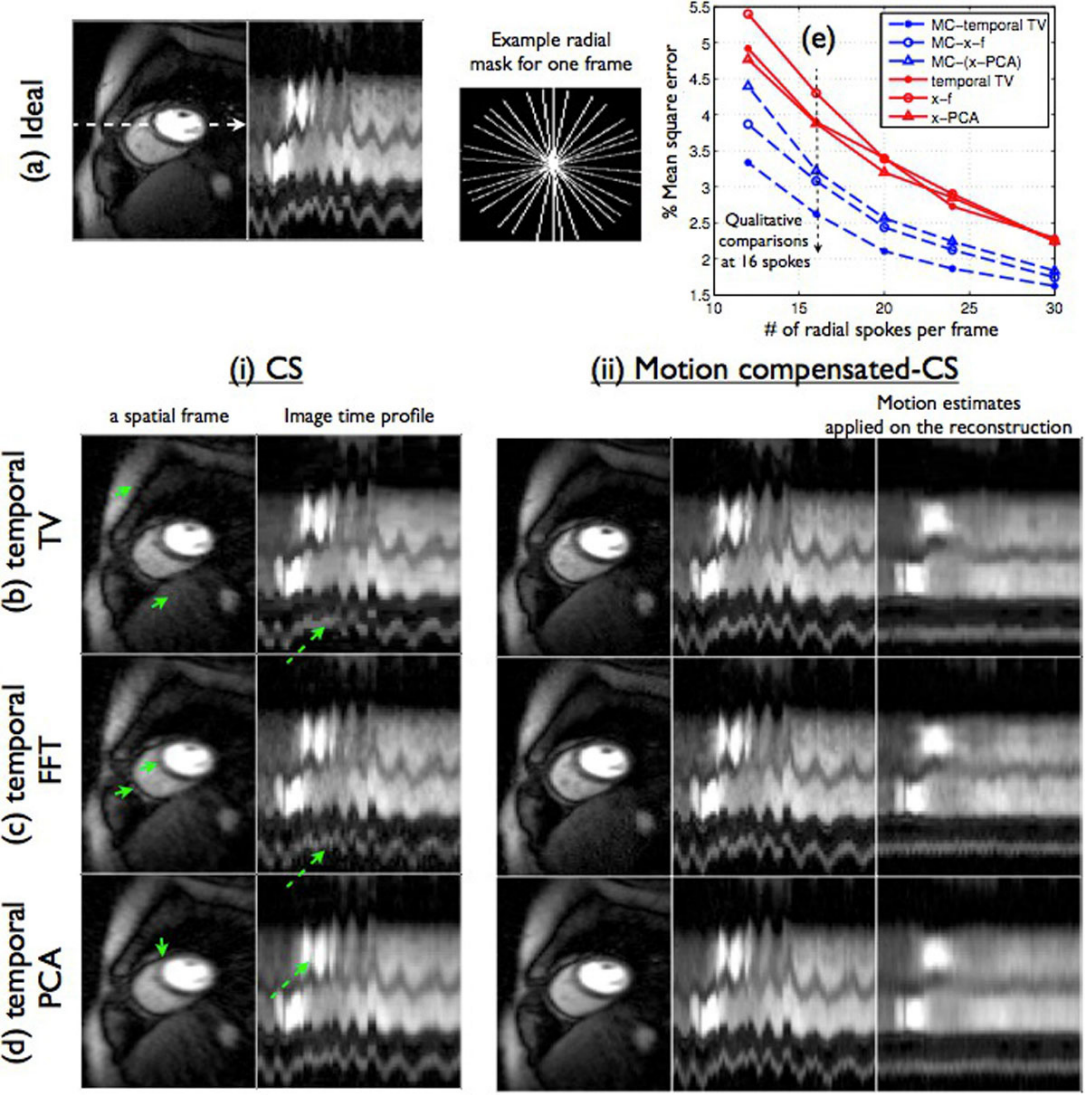
**Performance evaluation using retrospectively sampling on Cartesian data**: The compressed sensing (CS) reconstructions depicted motion related artifacts and temporal blurring (see arrows in (i)), while the proposed motion compensated CS images (MC-CS) were robust to these compromises. From (e), it can be seen that the percent mean square values were consistently lesser with the MC-CS schemes compared to their CS counterparts.

The MC-CS scheme jointly estimates the motion and the images using a variable splitting based optimization; this decouples the original problem into simpler sub problems. It iterates between the following steps until convergence: (a) temporal denoising/dealiasing of the deformed scene by promoting sparsity in either of (x-TV,x-f, x-PCA) domains, (b) motion estimation using nonrigid registration, (c) motion compensated reconstruction update. To validate, gated data was acquired using a saturation recovery FLASH sequence (TR/TE = 2.5/1 ms) on a Cartesian grid (90 × 190, 3 slices/beat). The data had motion due to inconsistent gating and breathing; additionally some integer shifts were added to amplify the motion (Figure 1). Retrospective undersampling of this data was done at various subsampling factors by using 30 to 12 spokes/frame. Prospective radially acquired data under free breathing, stress conditions were also considered from one subject (TR/TE = 2.6/1.2 ms, 72 rays/frame with uniform rotations across frames, 3 slices/beat). Single coil reconstructions were performed for this data by considering a subset of 24 rays from this data, which approximately followed the golden angle distribution.

## Results

**Figure 2:**
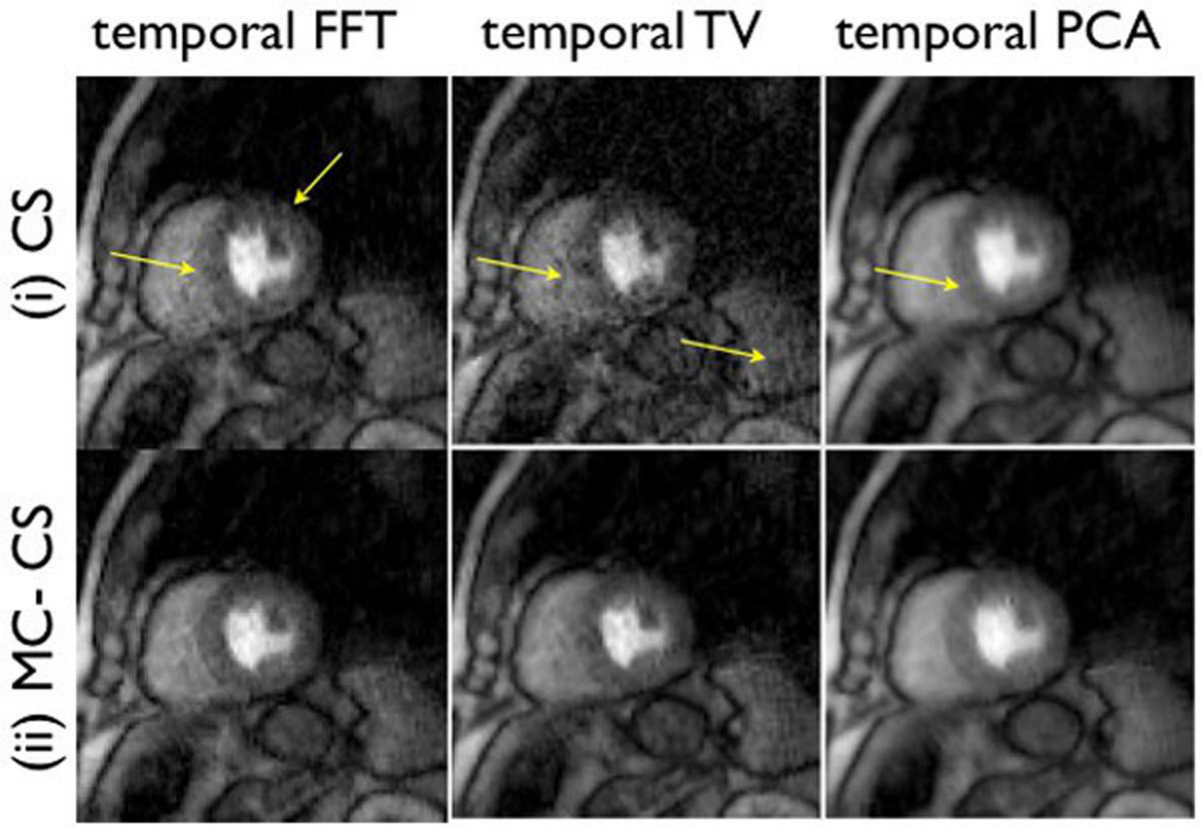
**Example image frames from a free breathing stress exam**. Reconstruction was performed using 24 rays/frame. The CS images showed motion artifacts in blurring and residual streaking as depicted in the top row, while the proposed MC-CS scheme was more robust to these compromises.

From Figure 1 and 2, it can be seen that the CS reconstructions depicted loss in temporal fidelity and motion related artifacts, while the corresponding MC-CS reconstructions were found to be robust to these artifacts.

## Conclusions

A motion compensated compressed sensing scheme has been demonstrated to reduce motion related artifacts in the context of accelerated myocardial perfusion MRI. The preliminary results in this work show promise; future validations on multiple patient scans are required to fully evaluate the method

## Funding

Grant support from NSF CCF-0844812, NSF CCF-1116067, NIH 1R21HL109710-01A1, and AHA 12 PRE11920052.

